# The impact of mixed-cultural speech on the stereotypical perception of a virtual robot

**DOI:** 10.3389/frobt.2022.983955

**Published:** 2022-11-18

**Authors:** David Obremski, Paula Friedrich, Nora Haak, Philipp Schaper, Birgit Lugrin

**Affiliations:** Human-Computer Interaction (HCI) Group, Department of Computer Science, University of Würzburg, Würzburg, Germany

**Keywords:** non-native accent, social robotics, intelligent virtual agents, stereotypes, mixed-cultural, culturally aware, socially interactive agents

## Abstract

Despite the fact that mixed-cultural backgrounds become of increasing importance in our daily life, the representation of multiple cultural backgrounds in one entity is still rare in socially interactive agents (SIAs). This paper’s contribution is twofold. First, it provides a survey of research on mixed-cultured SIAs. Second, it presents a study investigating how mixed-cultural speech (in this case, non-native accent) influences how a virtual robot is perceived in terms of personality, warmth, competence and credibility. Participants with English or German respectively as their first language watched a video of a virtual robot speaking in either standard English or German-accented English. It was expected that the German-accented speech would be rated more positively by native German participants as well as elicit the German stereotypes credibility and conscientiousness for both German and English participants. Contrary to the expectations, German participants rated the virtual robot lower in terms of competence and credibility when it spoke with a German accent, whereas English participants perceived the virtual robot with a German accent as more credible compared to the version without an accent. Both the native English and native German listeners classified the virtual robot with a German accent as significantly more neurotic than the virtual robot speaking standard English. This work shows that by solely implementing a non-native accent in a virtual robot, stereotypes are partly transferred. It also shows that the implementation of a non-native accent leads to differences in the perception of the virtual robot.

## 1 Introduction

Large efforts in research and development of socially interactive agents (SIAs) have lead to prototypes that are able to interact with humans multimodally and exhibit communication styles known from human-human interaction (Lugrin et al., 2021). Based on Lugrin (2021) we use the term *Socially Interactive Agents (SIAs)* as it includes virtually and physically embodied agents, which we reference as *Intelligent Virtual Agents* (IVAs) and *Social Robots* (SRs) respectively, when discussing only one of the potential embodiments. SIAs are nowadays being successfully used in domains such as health care or education, and managed to assist individuals and society.

Culture might not be the first aspect that comes into mind when implementing a SIA. It must be noted, however, that a SIA cannot be without culture, as the designer will subconsciously implement their own cultural background into the SIA, as they are the one’s judging on the SIA’s naturalness or appropriateness of appearance and behaviour (Lugrin and Rehm, 2021a). Thus, one line of research has focused on explicitly modelling culture-specific behaviours and to study culture-specific perceptions of SIAs (see Lugrin and Rehm, 2021a for an overview).

A slowly growing sub-area of research on culture and SIAs is on mixed-cultural SIAs. Considering the rising numbers in immigration, specifically interactions with mixed-cultural individuals have become an every day occurrence. We thus find research in this area of particular relevance to better understand the impact of mixed-cultural behaviours in SIAs, as well as potentially support mixed-cultural individuals in certain situations, and raise cultural diversity in virtual environments and games.

In order to implement and investigate the interaction with SIAs, specifically in a mixed-cultural context, it is necessary to first consider relevant research from the social sciences. When an individual migrates to a different country, certain cultural norms and language need to be adapted to. It is common to maintain certain behaviours and cultural norms of one’s home country even after having spent years in a host country, which makes non-native individuals oftentimes easily identifiable as foreign. This regularly leads to an individual being perceived as an outgroup member, as they show a different cultural background compared to the prevailing standard in the respective country. Tajfel et al. (1971) have discovered that whilst members of one’s ingroup are usually rated high on the dimensions warmth and competence (Fiske et al., 1999), members of an outgroup elicit ambivalent stereotypes, i. e. high ratings on one dimension and low ratings on another. Dragojevic and Goatley-Soan (2022) and Gluszek and Dovidio (2010) suggested that accented speech, specifically, which is regularly found in mixed-cultural individuals, can trigger stereotypes. In order to understand and possibly minimise those stereotypes, research is done on the perception of speech of persons with different cultural backgrounds. For instance, Dragojevic and Goatley-Soan (2022) investigated the attitudes towards English speakers with nine non-Anglo foreign accents and pointed out that some varieties, such as the German accent, were rated more favourably and easier to understand than others.

Before investigating the impact of mixed-cultural SIAs and their subsequent practical benefits in, for example, cultural training scenarios, culture-specific cues have to be implemented. There are several options to implement a mixed-cultural background in SIAs. Since speech is the primary channel of communication in human-human interaction (Nass and Brave, 2005), it is also very important when implementing (mixed-cultural) SIAs. Just as a SIA cannot be without a cultural background, intended or unintended, a SIA’s speech always has certain characteristics such as a perceived accent or an underlying emotion, even if the voice is designed to be as neutral as possible (Aylett et al., 2021). Given the amount of implicit information transmitted through speech, it is predestined to be carefully attuned to the intended cultural background of a SIA. Moreover, the SIA’s appearance and non-verbal behaviour and even the environment the SIA is presented in, can be developed and adapted depending on the culture that is to be depicted. However, for the implementation of mixed-cultural SIAs, speech is especially useful since for example a non-native accent is reliable identifier of a mixed-cultural person that moved to another country and had to learn a new language. In contrast to some of the other cultural cues, non-native speech is easily recognisable and hard to conceal.

This paper first provides an overview of the research efforts, regarding the implementation and perception of mixed-cultural appearance, speech and non-verbal behaviour, when investigating SIAs. Further, we present a study, investigating the perception of a virtual robot with a non-native accent. Finally, we give recommendations for future work with mixed-cultural SIAs and enculturated SIAs in general.

## 2 Survey on related work of implementing mixed-cultural SIAs

Culture can be implemented by manipulating external features (e.g., appearance and verbal behaviour) or internal features (e.g., assumptions or attitudes) of SIAs (Lugrin and Rehm, 2021b). Within this work we focus on the role of external features in order to implement convincing mixed-cultural SIAs. We consider work from the field of social robots and IVAs, since for the latter a larger body of work exists. However, due to hardware limitations, it must be noted that results from the research with IVAs might not always be transferable to social robots, especially in cases of subtle non-verbal behaviour. Culture-specific speech however, is easily adaptable for both IVAs and social robots and therefore plays a special role when designing mixed-cultural SIAs.

Based on Lugrin and Rehm (2021b) we use the term *enculturated* rather than *culturally aware*, because the latter implicitly entails that the SIA is self-reflective which is not the case in regard to previous work on the topic as well as the SIA used in our study. Culture-specific appearance, non-verbal and verbal behaviour can be used to implement enculturated SIAs with either a mixed-cultural background (consisting of multiple cultural identities) or a mono-cultural background (consisting of one cultural identity).

In the following section, previous research on the implementation and perception of mixed-cultural SIAs is presented, divided into the manipulation of appearance, non-verbal and verbal behaviour.

Since some forms of appearance and behaviour remain unchanged when adapting to a new culture, not all of them work as a mixed-cultural marker on their own. In these cases, research on the implementation and perception of mono-cultural appearance and behaviour is presented, since these cues, in combination with other culture-specific cues, can still be used to implement convincing mixed-cultural IVAs.

### 2.1 Mixed-cultural appearance for SIAs

For implementing mixed-cultural SIAs of a specific origin, adapting the SIA’s appearance can play an important part. However, there is no specific *mixed-cultural appearance* but rather a culture-specific appearance that appears in combination with certain behaviours, or contexts, that identify an individual as being mixed-cultural. A person migrating to another country, for example, might look the same as before, but adapt his or her behaviour based on the new cultural environment. Hence, the appearance might still be a valuable indicator of a person’s origin that, in a given environment, suggests a mixed-cultural background. Therefore, when developing and investigating SIAs, it is important to focus on a accurate implementation of visual cultural cues.

The following section provides an overview of the research efforts, exploring the development of culture-specific appearance and the perception of enculturated SIAs.

#### 2.1.1 Physical appearance

When implementing an SIA, different aspects about its appearance can be adapted, such as skin colour, hair colour or other physical features like shape of the face or body. Depending on the cultural background that is to be depicted, physical characteristics are adjusted accordingly, like, for instance, in the Tactical Language and Culture Training System by Johnson and Valente (2009) to convey a culturally congruent SIA. McDonnell and Mutlu (2021) and Hayes-Roth et al. (2002) have pointed out that an agent’s appearance provides information about the agent’s characteristics, such as gender, age, socioeconomic background and culture. They furthermore discuss the differences in implementation when developing virtual agents which allow a variety of specific design options, whereas the development of physical agents requires the use of ambiguous physical features, for instance, features such as (interchangeable) hair and lips. The appearance of SIAs additionally affects their credibility and communicates cultural cues to the users prior to the SIA’s use of speech or non-verbal behaviour (Hayes-Roth et al., 2002).

Aylett et al. (2014) implemented the MIXER system which aims at raising children’s cultural sensitivity by letting them play a game with virtual characters with different culture-specific appearances that exhibit what is initially perceived as unfair behaviour but later explained being caused by a different set of rules the virtual characters follow.

To investigate whether the positive effects of mirroring and similarity on outgroup trust are transferable to human-agent-interaction, (Tamborini et al., 2018) developed virtual agents of either Caucasian African-American background by altering their visual features, such as skin colour. Participants in the experiment were asked to play a dance game with a virtual agent and were told that they would be rated on their level of synchrony with the virtual agent and their dance skills. For Caucasian participants, the two conditions entailed either dancing with a Black agent (outgroup) or dancing with a Caucasian agent (ingroup). This was reversed for African-American participants. As a result they could firstly show that participants successfully identified the enculturated virtual agents as either similar (ingroup agent) or different (outgroup agent) to their own race. Additionally, they were able to show that with increased synchrony in dance moves between the participant and the virtual agent, there was also higher outgroup trust in the participants. This highlights the effects of culture-specific visual alterations in SIAs on real-life outgroup trust.

In order to investigate real-life bias in virtual scenarios, (Rossen et al., 2008) created two IVAs, one with a light skin-tone and dark-blond hair as well as another agent with a dark skin-tone and dark-brown hair, to elicit culture-specific categorisation in the user. In the experiment, medical students were asked to carry out 10-min patient interviews with the virtual agents, which included assessment of previous illnesses and current health problems of the patients. The results showed higher levels of empathy in the participants in the condition with the light skin-tone virtual agent compared to the condition with the dark skin-tone agent. Additionally, using the Implicit Association Test, the authors were able to show a significant correlation of real-world bias and the bias identified towards the virtual character. Such research further validates the use of virtual characters in cultural trainings in the future. There have been many studies on culture-specific appearance and the resulting effects on the users’ perception of the SIAs, showing that alterations in appearance were successful in conveying the SIA as foreign.

#### 2.1.2 Clothing

Whilst the studies presented above have implemented culture-specific appearance in SIAs, they have mostly focused on changes in skin-tone and facial as well as body features and its effects on the user. However, the additional implementation of culture-specific clothing can contribute to a more precise development of enculturated SIAs. Lugrin et al. (2018c), for instance, investigated Adapted Foreigner-directed Communication with virtual agents and developed two female agents that were both of different ethnical backgrounds, one with prototypical Northern European appearance and one with prototypical Arabian appearance. Whilst the Northern European virtual agent was rather tall, with rose-white skin and blond to ginger hair, the Arabic virtual agent was developed to be less tall with a gracile body form as well as yellow-white skin. Additionally, a head scarf was added to the clothing of the Arabic virtual agent. Hasler et al. (2014) took a similar approach by developing a male Palestinian virtual agent, in order to investigate contact with an outgroup member in a virtual scenario. To convey a Palestinian background, the virtual agent was wearing dark-coloured jeans and a long-sleeve white button-down shirt. Additionally, the virtual agent wore white headgear.

### 2.2 Mixed-cultural non-verbal behaviour for SIAs

Similar to a person’s appearance, its non-verbal behaviour can help identify it as a mixed-cultural individual, when combined with other modalities. Several studies have investigated the non-verbal behaviour native speakers display when talking to non-native speakers (e.g., Tellier and Stam, 2010; Azaoui, 2013), as well as the non-verbal behaviour mixed-cultural individuals themselves exhibit. Research analysing the non-verbal behaviour of non-native speakers has revealed, that mixed-cultural individuals tend to display the same non-native behaviour when talking in their non-native language as when talking in their native language (Busà and Rognoni, 2012). Therefore, researchers mostly implement culture-specific non-verbal behaviour based on the SIAs intended *original* culture (e.g., Lugrin et al., 2018c) in order to design convincing mixed-cultural SIAs. Research, investigating how culture-specific non-verbal behaviour observed in human-human interaction transfers to human-agent interaction has investigated gestures (e.g., Lugrin et al., 2018a; e) as well as facial expressions (e.g., Koda et al., 2010; Koda and Ruttkay, 2017). The following section presents work on the culture-specific implementation of non-verbal behaviour for SIAs, focusing on gestures, as they are more universally adaptable for social robots, since they often have limited facial expressions.

Endrass et al. (2011) conducted a study, where they implemented different culture-specific non-verbal behaviours for IVAs to either match the German or the Japanese culture. In the subsequent evaluation with German and Japanese participants they were able to show, that the participants tended to prefer the IVA that displayed the non-verbal behaviour that matched their own cultural background.

Obaid et al. (2012) conducted a similar study in augmented reality to investigate how participants react when an IVA displays non-verbal behaviour that is not congruent with their own cultural background. With Arabs and Germans as participants they found that when the non-verbal behaviour of the IVA is not matching the participant’s respective background, they show higher physiological arousal towards these IVAs.

Jan et al. (2007) presented a computational model, based on literature on the topic, for implementing culture-specific gaze, proxemics and inter-turn pause length in IVAs. To evaluate, whether the culture-specific behaviours regarding the three dimensions are perceived as realistic by people of the implemented culture, the authors conducted a study. During the study, the participants, who were either American, Mexican, or Arabic, watched simulations of multiple IVAs conducting a silent conversation showing the previously generated non-verbal behaviour of each of the cultures respectively. The results show, that the three dimensions gaze, proximity and inter-turn pause length were only partly sufficient to trigger the impression of a realistic cultural behaviour. The authors note that apart from these three dimensions, the IVAs’ clothes and their gestures could have played a role in the users’ perception of them being a realistic representation of their culture.

Investigating the culture dependent dimensions smile, head position and eye contact, on Syrian participants’ perception of a social robot, (Lugrin et al., 2018b) conducted a study in which participants were asked to rate a social robot that either showed Arabic or German traits of the three dimensions. Syrian participants showed a partial preference for the robot showing Arab traits during the interaction. However, regarding perceptual ratings such as trust, anthropomorphism, animacy, likeability, perceived intelligence, and perceived safety no significant differences were observed between the two robots.

Cross cultural research in the field of social robotics conducted by Eresha et al. (2013) has shown, that Germans and Arabs differ in terms of which interpersonal distance they prefer when interacting with a robot. In one static and one dynamic setting the authors were able to show that the users tend to prefer proxemic behaviour that reflects their own cultural background when interacting with a social robot. In line with previous results from human-human interaction, Arabs preferred a closer interpersonal space in multi-party conversations compared to Germans.

### 2.3 Mixed-cultural speech for SIAs

Speech plays a crucial role when designing mixed-cultural SIAs. Speech does not only act as a way of explicit communication, but also as a way of transferring implicit information about the speaker, such as the emotional state, the state of health, its geographical or social background (Aylett et al., 2021). In contrast to the SIA’s appearance and its non-verbal behaviour, which itself in most cases only gives cues about the SIA’s intended *original* culture, speech has the ability to trigger the impression of a non-native speaking SIA, hence a mixed-cultural SIA (Obremski et al., 2021). Other than for non-verbal behaviour or appearance such as culture-specific clothing, it is not as easy for a non-native speaker to adapt his verbal behaviour to fully integrate into a new cultural environment. Therefore, speech is particularly suited as a cultural cue for implementing mixed-cultural SIAs, since characteristics like grammatical mistakes or non-native accents inevitably occur in a mixed-cultural individual’s speech.

#### 2.3.1 Grammatical mistakes

One indication of a speaker’s origin being different than their current abode is the occurrence of grammatical mistakes in someone’s speech. While specific deviations from the grammar of a language’s standard variety can be attributed to regional dialects (Hughes et al., 2013), others may identify the speaker as a non-native speaker of this language (Csehó, 2009; Lee and Seneff, 2008; Ting et al., 2010; Richards, 1971). Whereas some research has implemented SIAs with grammatically incorrect speech in combination with other cultural cues (Lugrin et al., 2018d), work where the systematic implementation of grammatical mistakes with SIAs is investigated, is rare. We conducted two studies (Obremski et al., 2019; 2021) to identify, how grammatical mistakes typical for non-native speakers of German and English can be induced in an IVA’s speech, and below which threshold of language proficiency the IVA is consistently perceived as a non-native speaker of either German or English.

For the German study we induced two grammatical mistakes typically made by non-native speakers of German, which could be induced automatically. One such mistake is misplacing words in a sentence, the other is using the infinitive form for verbs that should be conjugated (Csehó, 2009). The results of the German study suggest, that when misplacing at least 10% of the words in a text or using 25% of the verbs that should be conjugated in their infinitive form, an IVA is perceived as a non-native speaker of German.

For the English study, we also implemented two grammatical mistakes, which are typical for non-native speakers of English. While the wrong use of the infinitive form is also a common mistake among non-native speakers of English, the misplacing of words is not as common. Therefore we implemented the more frequent mistake of omitting prepositions and articles in addition to the infinitive mistake (Lee and Seneff, 2008; Ting et al., 2010; Richards, 1971). The results of the English study show that when the IVA omits at least 50% of the articles or prepositions of a text or when it uses at least 50% of the verbs that should be conjugated in their infinitive form, an IVA is perceived as a non-native speaker of English. These two studies show, that the impression of a non-native speaking IVA can be achieved by inducing grammatical mistakes at certain rates. An additional question in the English study, regarding the IVA’s mother tongue after labelling it as a non-native speaker, revealed that solely based on grammatical mistakes, the participants were not able to assign a certain mother tongue to the IVA.

#### 2.3.2 Non-native accent

In contrast to grammatical mistakes, accent has the potential to give more specific information about the speaker’s geographical origin and to elicit stereotypes associated with that accent (Aylett et al., 2021; Ikeno and Hansen, 2007; Coupland and Bishop, 2007). While there are various approaches in social robotics focusing on the recognition of non-native user speech (Moussalli and Cardoso, 2017; Mubin et al., 2012; Irfan et al., 2020) there is very little research using non-native accents on the agent’s side. Three studies in total were conducted by Dehghani et al. (2011) and Khooshabeh et al. (2017), investigating the perception of English-speaking IVAs with an Iranian or Chinese non-native accent and whether interacting with these IVAs can lead to cultural frame switching within participants that share the IVA’s mixed cultural background. The authors showed that interacting with the IVAs with the non-native accents shifts the participants’ interpretative lens towards their *original* culture (Iranian or Chinese, respectively). Furthermore they recognised the Chinese accent as *more Asian* and the Iranian accent as *more Middle Eastern*.

#### 2.3.3 Regional accent

Since research on non-native accents in the field of SIAs is rare, it is worth taking a look at the adjacent research field of regional accents with SIAs. To evaluate, which accent users would like a social robot to have, Torre and Le Maguer (2020) conducted an online survey with over 500 British participants. Participants were asked to indicate to which accent they were exposed to most while growing up and what accent they would like a robot to have. Most of the participants indicated that they would prefer the robot to have a Standard Southern British English accent, followed by an Irish accent. Contrary to the authors’ expectations and the similarity attraction principle, participants did not tend to name their own accent as a preferred accent for the robot.

Investigating how a regional accent and dialect impacts the perception of a social robot, we conducted a study measuring the perceived competence, as well as the likeability and social skills of a robot speaking with regional language varieties (Lugrin et al., 2020). The results show that the robot speaking with standard German was perceived as more competent than the robot speaking with regional language varieties. The participants rated the robot with the regional language varieties higher on perceived competence, likeability and social skills the more participants spoke the respective dialect themselves.

In the field of IVAs various studies on the perception of regional language varieties have been conducted. In a study by Torre et al. (2015) they let participants play an investment game with an IVA with either a Standard Southern British English accent or a Liverpool English accent, to test if the initial trust attribution changes over time, depending on the IVA’s accent and behaviour. When the IVA acted generously, the participants invested more when it spoke with the Standard Southern British English accent. When it acted mean, the participants at first invested more when it spoke with the Standard Southern British accent. However, after a few rounds of the IVA acting mean the investments in it when it spoke with the Standard Southern British accent dropped to a higher degree, compared to the IVA with the Liverpool English accent. The authors interpret these results as a stronger negative reaction towards unjust behaviour in combination with a prestigious accent.

Krenn et al. (2012) investigated how an invisible tour guide is perceived when speaking with either synthetically generated standard Austrian or dialectal Viennese. Their results show that the dialectal Viennese voice was rated higher on items for naturalness and relaxedness while the standard Austrian voice was rated higher on items for competence and intelligence. Building on this study we investigated how an embodied IVA is perceived when speaking with different regional language varieties (Krenn et al., 2014). The comparison showed that the IVA was rated as more extroverted when speaking with a synthetically generated Viennese or standard Austrian dialect when compared to speaking with the German standard variety.

Another study conducted by Kühne et al. (2013) found that IVAs speaking with dialectal German are rated as more likeable compared to IVAs speaking with standard German. The results of their study furthermore show that participants used more dialectal words when speaking with the IVA, which spoke with dialectal German.

### 2.4 Conclusion and objective

While the congruent implementation of multiple cultural cues at once has been shown to generate the impression of a convincing mixed-cultural SIA (e.g., Lugrin et al., 2018c), it is still important to investigate whether isolated cultural cues can lead to a similar perception, resulting in a more flexible and cost effective design. Even though humans are used to experience an interplay of various cultural cues in their interlocutors, some isolated cultural cues might work on their own, since the human brain is known to fill in missing information to make sense of the world (e.g., Kashino, 2006).

Since most of the research conducted on the topic is in the field of IVAs, there is still a deficit in work on mixed-cultural social robots. Specifically, research on mixed-cultural speech and synthetic non-native accents is rather rare. While IVAs have the advantage of easily modifiable appearance, for social robots it is rather difficult, and basically limited to attached accessories like stickers (as for example done by Häring et al., 2014). Similarly, non-verbal behaviour can subtly be animated with IVAs and culture-specific variation can be easily displayed, while social robots are more restricted in terms of subtle animations due to hardware limitations. Speech on the other hand, can be easily adapted for both IVAs and social robots and therefore has the potential to act as a cultural marker for both.

The following study investigates, how mixed-cultural speech in form of a German accent impacts the perception of a virtual robot within German and English native speakers. The results of this study can be helpful for future work with mixed-cultural IVAs, as well as social robots because they focus on easily adaptable synthetically generated mixed-cultural speech, regardless of the SIA’s appearance and non-verbal behaviour.

Based on the theoretical background on the rating of ingroup members in the dimensions of warmth and competence (Fiske et al., 1999), the potential of speech eliciting stereotypes (Aylett et al., 2021; Ikeno and Hansen, 2007; Coupland and Bishop, 2007), as well as the German stereotypes of being *hard working* and *trustworthy* (Kohut et al., 2012; Kohut et al., 2013), we formulate the below hypotheses. Previous work has shown that different personality traits can act as predictors for the strength of the non-native accent a person retains when learning a new language (Zárate-Sández, 2017). It is, however, not clear whether recipients of non-native accents attribute the respective personality traits to the speaker. Therefore, to further investigate, whether the use of a synthetic non-native accent leads to other effects concerning the virtual robot’s perceived personality, we formulate an additional research question.

H1: German native speakers rate the virtual robot speaking with a German accent higher on perceived warmth, competence, and sympathy.

H2: The virtual robot speaking with a German accent triggers German stereotypes and is therefore generally rated higher on credibility and conscientiousness.

RQ: Does the virtual robot’s non-native accent affect its perceived personality in terms of extroversion, agreeableness, neuroticism, and openness?

## 3 User study

To investigate, how a virtual robot, speaking German-accented English in comparison to standard English, is perceived by German or English native speakers, we conducted an online experiment. The experiment was conducted online due to the ongoing pandemic.

### 3.1 Stimuli generation

For the online experiment two videos had to be produced that show a virtual robot in a virtual environment, holding a monologue, speaking English with either German-accented English or standard English.

#### 3.1.1 Design of the virtual robot

Based on a cooperation with the German company Infosim^1^ their company mascot, a 2D-figure resembling a robot was used to model a 3D virtual robot. As designed by Infosim, the virtual robot wore a helmet in the style of a construction worker. The 3D modelling was done using Blender^2^. The robot’s design included a sketched mouth but no movable lips, so the robot’s voice appeared to be originating out of hidden speakers built into the robot. The virtual robot was rigged to be able to move its legs and arms to enable the implementation of basic gestures. To accompany the monologue held by the virtual robot, simple gestures such as waving and various beat-gestures were implemented. A virtual robot, despite its non-human appearance, might unintentionally trigger human-like associations within the user through for example specific clothing or colouring (Carolus et al., 2018; Trovato et al., 2016). In contrast to a humanoid SIA however, a virtual robot allows for a more isolated exploration of speech as a cultural cue, since the cultural cues that it may trigger are not explicit.

#### 3.1.2 Design of the environment

**FIGURE 1:**
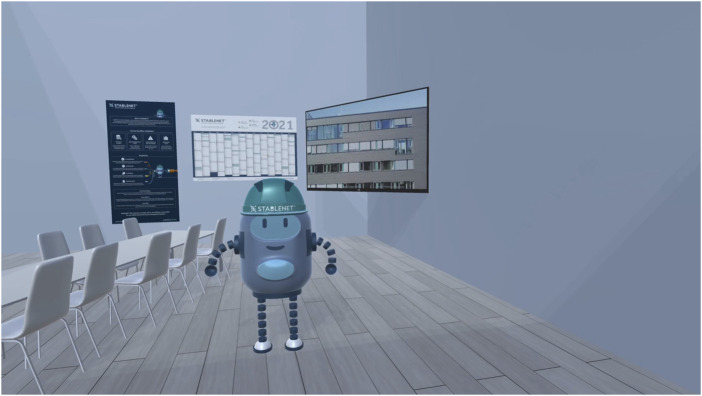
Screenshot of the virtual robot used in this study.

A virtual environment was created using Unity (Version 2020.1.0b4)^3^ for the virtual robot to be placed in. The environment resembled a neutral meeting room, containing a table and several chairs, as well as a television screen in the background. During the virtual robot’s monologue, the screen showed an image video provided by Infosim, which contained visualisations of the virtual robot’s spoken text. Figure 1 shows a screenshot of the robot in the virtual environment.

### 3.1.3 Generation of speech

The text for the virtual robot’s monologue consisted of 277 words and was part of a product presentation by Infosim. In the text, a network and service management platform gets advertised. The text contained mostly technical descriptions and no obvious cultural cues or references to specific locations. The text-to-speech engine Amazon Polly^4^ was used to generate synthetic speech files. The speech file containing the standard English version of the text was generated using the synthetic voice named “Joey” provided by Amazon Web Services. For the standard English version only minor modifications were made using techniques like splitting words to increase the intelligibility of certain words. The English speech file with the German-accented English was generated by using the German text-to-speech system by Amazon Polly with the synthetic voice named “Hans.” The speed of the generated speech was set to 90% using SSML to better match the speech with the standard English accent. Subsequently, the English text was inserted and enclosed by the SSML-tag *lang xml:lang =* “*en-US*,*”* which, according to Amazon Web Services, results in an English pronunciation with a German accent. This is accomplished by applying some pronunciation rules from the English language and others from the German language. Analogously to the standard English speech, some words were split or slightly modified for increased intelligibility. Furthermore, certain words were modified to resemble a more realistic German accent. Since the authors were native German speakers, they were able to identify words that did not match the desired German-accented pronunciation and to improve the pronunciation by altering the writing of the words. While not all adaptions could be summarised to rules, the alternative writings for the (se), this (zis), and with (wif) were used every time the words appeared in the text. To specifically investigate the effect of the non-native accent on the perception of the virtual robot, no grammatical mistakes were inserted in the text in either condition. The final speech files were exported as.mp3 and imported into the Unity scene to be played back by the robot. For the final stimuli, two videos were exported. One showing the virtual robot holding the monologue with standard English speech and one showing the virtual robot holding the monologue with German-accented English speech.

### 3.2 Study design and procedure

A 2 × 2 between subjects design was used, resulting in either German or English native speakers, watching a video of the virtual robot speaking with either standard English or German-accented English. Before watching the randomly assigned video, participants filled in demographic data. After watching the video, the dependent variables were assessed in the same questionnaire. To assess whether the English speaking participants had any knowledge of the German language without biasing their answers to the dependent variables, an additional question appeared at the end of the questionnaire.

### 3.3 Participants

Participants were acquired partly *via* email and partly using the established recruitment platform Prolific^5^. Participants were pre-screened to be either native speakers of German or English. Overall 218 participants took part in the study. In the group of native English speakers (*n* = 119) the mean age was 30.36 (SD = 12.41) with 73 female, 42 male and 4 participants of diverse gender. For the group of German native speakers (*n* = 99) the mean age was 35.71 (SD = 15.61) with 49 female, 49 male and 1 participant of diverse gender. After the assessment of the dependent variables, English speaking participants were asked: *How long have you been speaking German*. Participants were able to select between the following answers: *I don’t speak German* (*n* = 99)*, less than 1 year* (*n* = 6)*, 1–3 years* (*n* = 5)*, 3–5 years* (*n* = 1)*, more than 5 years* (*n* = 4)*, mother tongue (bilingual)* (*n* = 4).

### 3.4 Measures

#### 3.4.1 Intelligibility and accent

The participants were asked the following question to indicate how well they were able to understand the virtual robot: *How well could you understand the intelligent virtual agent?* on a five-point likert scale from 1 (not at all) to 5 (very well). To evaluate, whether the participants perceived the virtual robot to have an accent they were asked the following question: *Would you say that the intelligent virtual agent (IVA) had an accent?* ***yes or no***. If they answered that the virtual robot did have an accent, an additional open question appeared, asking to name the accent they would assign to the virtual robot. In order to assess the participant’s own accent, they were asked if they believe they speak with an accent themselves (yes or no) and if so, which one it is (as an open question).

#### 3.4.2 Warmth and competence

The scale on warmth and competence by Fiske (2018) was used to assess the respective variables. The perceived warmth of the virtual robot was assessed by indicating on a scale from 1 (not at all) to 5 (totally), whether the virtual robot appeared *warm, friendly, good-natured, sincere, well-intentioned,* and *trustworthy*. The perceived competence of the virtual robot was assessed by indicating on a scale from 1 (not at all) to 5 (totally), whether the virtual robot appeared *competent, confident, capable, intelligent, skillful,* and *efficient*.

#### 3.4.3 Credibility and sympathy

To assess the German stereotype of trustworthiness we measured the perceived credibility of the virtual robot, using the scale from Ohanian (1990). Participants were asked to indicate their agreement with the IVA being *dependable, honest, reliable, sincere,* and *well-intentioned* from 1 (not at all accurate) to 5 (completely accurate).

To assess the participants’ sympathy for the virtual robot, the respective scale by Reysen (2005) was used. It consists of 11 items, which participants were asked to indicate their agreement with, reaching from 1 (not at all accurate) to 5 (completely accurate). It contains statements like “The IVA is friendly” or “I would like to be friends with this IVA.”

#### 3.4.4 Big-Five Inventory

The virtual robot’s perceived personality was assessed with the BFI-10 scale developed by Rammstedt et al. (2013), which is a short version of the Big-Five Inventory that consists of two items per dimension. It includes the dimensions *Extroversion, Agreeableness, Conscientiousness, Neuroticism,* and *Openness*. The participants had to indicate their agreement with ten statements, reaching from 1 (not at all accurate) to 5 (completely accurate). This questionnaire includes statements like “I see this IVA as someone who tends to be lazy” for *Conscientiousness* or “I see this IVA as someone who gets nervous easily” for *Neuroticism*. To assess the German stereotype of being *hard working*, the dimension *Conscientiousness* was used.

## 4 Results

For all subsequent analyses four participants were excluded from the group of English native speakers because they also reported native language proficiency in German. Descriptive data for all measures is presented in Table 1.

**TABLE 1:** Means for questionnaire measures for the respective groups and conditions. SDs in parentheses.

Scale	English native speakers		German native speakers	
	Standard English	German-accented	Standard English	German-accented
Intelligibility	3.71(0.89)	2.83(0.86)	3.58(0.76)	2.73(0.84)
Warmth	3.12(0.86)	3.25(0.87)	2.89(0.73)	2.63(0.82)
Competence	3.61(0.94)	3.60(0.80)	3.22(0.86)	2.54(0.73)
Extroversion	2.73(0.86)	2.63(0.75)	2.62(0.73)	2.64(0.75)
Agreeableness	3.35(0.66)	3.35(0.76)	3.02(0.68)	3.21(0.65)
Conscientiousness	3.98(0.68)	3.98(0.76)	4.01(0.84)	3.62(0.81)
Neuroticism	2.26(0.75)	2.46(0.71)	2.03(0.83)	2.29(0.78)
Openness	2.29(0.79)	2.46(0.83)	1.97(0.84)	2.05(0.87)
Credibility	3.23(0.91)	3.48(0.68)	3.17(0.97)	2.63(0.82)
Sympathy	2.48(0.66)	2.55(0.68)	2.19(0.60)	1.99(0.72)

Two Chi squared tests were calculated to investigate the participants’ perception of the virtual robot’s accent independent of the participants’ mother tongue. The first Chi squared test revealed a significant association of the virtual robot’s accent and the participants’ perception of it having an accent *χ* ^2^(1) = 85.08, *p* < .001. Within the German-accented English condition, 92.3% of the participants perceived the virtual robot to have an accent, while in the standard English condition only 32.2% of the participants did.

The second Chi squared test was calculated to see, whether the participants were able to assign the correct accent to the IVA (hence, *English* in the standard English condition and *German* in the German-accented English condition). The test revealed a significant association between the correctness of the assigned accent and the virtual robot’s accent *χ* ^2^(1) = 9.13, *p* = .003. Within the German-accented English condition, 77.7% of the participants recognised the accent as German, while in the standard English condition 92.2% of the participants recognised the accent as English.

To test if the accent affected intelligibility, we conducted a 2 × 2 ANOVA with the factors participants’ mother tongue (German native speakers, English native speakers) and virtual robot’s accent (standard English, German-accented English) for the questionnaire item (5-point Likert scale) on how easy it was to understand the agent. We found a significant main effect of the virtual robot’s accent (*F*(1, 209) = 2129.39, *p* = .014, $\eta_{p}^{2}=1$ ), with better intelligibility for the virtual robot speaking standard English. The virtual robot speaking standard English was understood significantly better than the virtual robot speaking German-accented English. There was no significant main effect of participants’ mother tongue (*F*(1, 209) = 35.11, *p* = .106, $\eta_{p}^{2}=.97$ ) and no interaction (*F*(1, 209) = 0.03, *p* = .872, $\eta_{p}^{2}<.01$ ).

A 2 × 2 MANOVA with the factors participants’ mother tongue (German native speakers, English native speakers) and virtual robot’s accent (standard English, German-accented English) was conducted for warmth and competence. Multivariate tests indicate a significant main effect of participants’ mother tongue (*F*(2, 208) = 20.22, *p* < .001, $\eta_{p}^{2}=.16$ ), of the virtual robot’s accent (*F*(2, 208) = 4.68, *p* = .009, $\eta_{p}^{2}=.05$ ), and a significant interaction (*F*(2, 208) = 4.22, *p* = .016, $\eta_{p}^{2}=.04$ ). We conducted post-hoc univariate ANOVAs to investigate the significant effects. For warmth we found a significant main effect of participants’ mother tongue (*F*(1, 209) = 14.01, *p* < .001, $\eta_{p}^{2}=.06$ ). English native speakers perceived the virtual robot in general as warmer than German native speakers. There was no significant main effect of the virtual robot’s accent (*F*(1, 209) = 0.18, *p* = .609, $\eta_{p}^{2}<.01$ ) and no interaction (*F*(3, 209) = 2.02, *p* = .086, $\eta_{p}^{2}=.01$ ).

**FIGURE 2:**
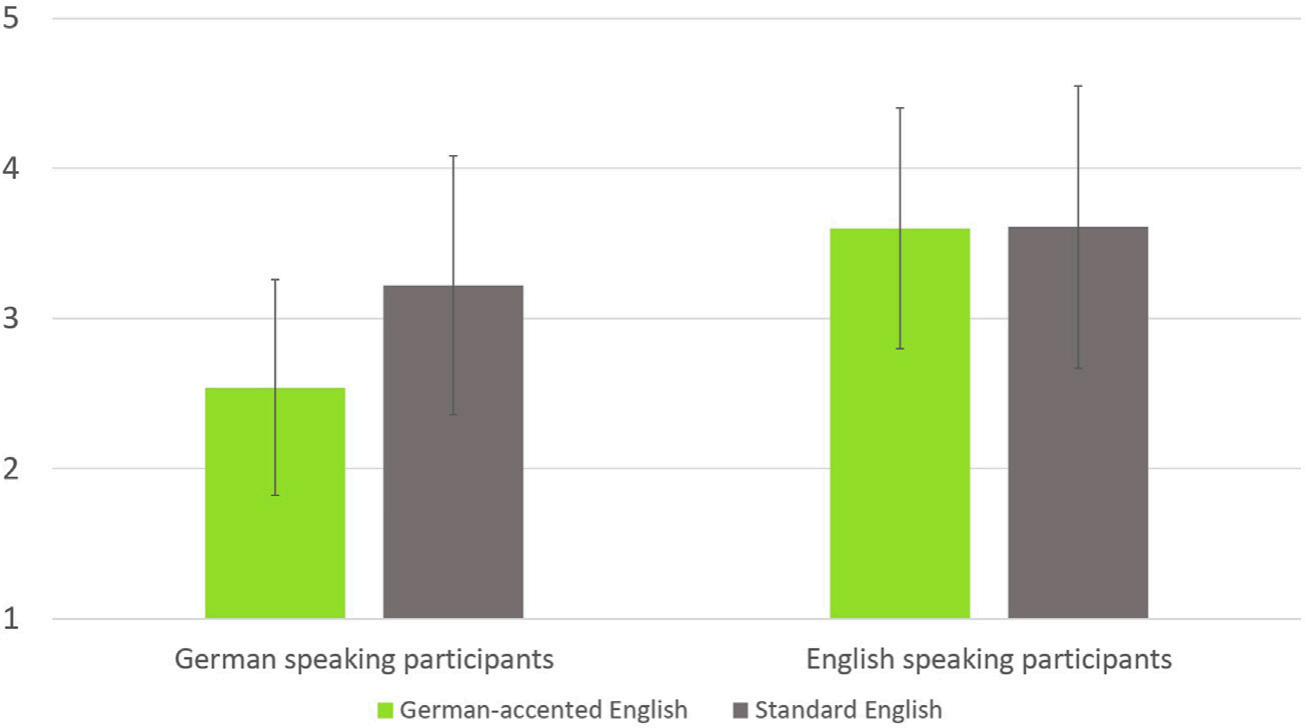
The results for perceived competence of the virtual robot in the different conditions. Error bars show the standard deviations.

For competence we found a significant main effect of participants’ mother tongue (*F*(1, 209) = 39.55, *p* < .001, $\eta_{p}^{2}=.16$ ). English native speakers perceived the virtual robot in general as more competent than German native speakers. The virtual robot’s accent also had a significant main effect (*F*(1, 209) = 8.93, *p* = .003, $\eta_{p}^{2}=.04$ ). The virtual robot speaking standard English was perceived as more competent than the virtual robot speaking German-accented English. The interaction was also significant (*F*(1, 209) = 8.24, *p* = .005, $\eta_{p}^{2}=.04$ ). We found nearly identical values for competence on both accents in the English native speakers and lower values for the German accent in the German native speakers. Figure 2 shows the results for the virtual robot’s perceived competence.

A 2 × 2 MANOVA with the factors participants’ mother tongue (German native speakers, English native speakers) and virtual robot’s accent (standard English, German-accented English) was conducted for all five subscales of the BFI (extroversion, agreeableness, conscientiousness, neuroticism, openness). Multivariate tests indicated a significant main effect of participants’ mother tongue (*F*(5, 205) = 4.64, *p* < .001, $\eta_{p}^{2}=.10$ ). The main effect of the virtual robot’s accent was not significant (*F*(5, 205) = 1.51, *p* = .188, $\eta_{p}^{2}=.04$ ), and there was no significant interaction (*F*(5, 205) = 1.25, *p* = .286, $\eta_{p}^{2}=.03$ ). We conducted post-hoc univariate ANOVAs to investigate the significant effects (see Table 2). We found a significant main effect of mother tongue for agreeableness with nearly identical values for both accents in the English native speakers and lower values for the German accent in the German native speakers, but overall higher values for the English native speakers. English native speakers perceived the virtual robot in general more agreeable than German native speakers. For openness there was a significant main effect of mother tongue with higher values for both accents in the English native speakers group. English native speakers perceived the virtual robot in general as more open than German native speakers. There was a significant main effect of robot accent on neuroticism with overall higher values for the German-accented English. The virtual robot speaking speaking German-accented English was perceived as more neurotic than the virtual robot speaking standard English. There were no other significant main effects or interactions.

**TABLE 2:** Main effects and interactions for post-hoc analysis of the BFI scales.

BFI subscale	Factor participants’ mother tongue	Factor virtual robot’s accent	Interaction
Extroversion *F*(1,209)	*F* = 0.24, *p* = 0.623, $\eta_{p}^{2}$ = 0.00	*F* = 0.13, *p* = 0.722, $\eta_{p}^{2}$ = 0.00	*F* = 0.33, *p* = 0.569, $\eta_{p}^{2}$ = 0.00
Agreeableness *F*(1,209)	*F* = 5.92, *p* = 0.016, $\eta_{p}^{2}$ = 0.03	*F* = 1.06, *p* = 0.305, $\eta_{p}^{2}$ = 0.01	*F* = 1.07, *p* = 0.302, $\eta_{p}^{2}$ = 0.01
Conscientiousness *F*(1,209)	*F* = 2.33, *p* = 0.128, $\eta_{p}^{2}$ = 0.01	*F* = 3.25, *p* = 0.073, $\eta_{p}^{2}$ = 0.02	*F* = 3.42, *p* = 0.066, $\eta_{p}^{2}$ = 0.02
Neuroticism *F*(1,209)	*F* = 3.69, *p* = 0.056, $\eta_{p}^{2}$ = 0.02	*F* = 4.77, *p* = 0.030, $\eta_{p}^{2}$ = 0.02	*F* = 0.06, *p* = 0.804, $\eta_{p}^{2}$ = 0.00
Openness *F*(1,209)	*F* = 10.23, *p* = 0.002, $\eta_{p}^{2}$ = 0.05	*F* = 1.22, *p* = 0.271, $\eta_{p}^{2}$ = 0.01	*F* = 0.16, *p* = 0.693, $\eta_{p}^{2}$ = 0.00

For the dependent variables credibility and sympathy we also conducted a 2 × 2 MANOVA with the factors participants’ mother tongue (German native speakers, English native speakers) and virtual robot’s accent (standard English, German-accented English). Multivariate tests indicated a significant main effect of participants’ mother tongue (*F*(2, 208) = 11.52, *p* < .001, $\eta_{p}^{2}=.1$ ), but there was no significant main effect of the virtual robot’s accent (*F*(2, 208) = 0.74, *p* = .477, $\eta_{p}^{2}=.01$ ). However, the interaction was significant (*F*(2, 208) = 5.87, *p* = .003, $\eta_{p}^{2}=.05$ ). We conducted post-hoc univariate ANOVAs to investigate the significant effects. For credibility we found a significant main effect of participants’ mother tongue (*F*(1, 209) = 15.08, *p* < .001, $\eta_{p}^{2}=.07$ ). English native speakers perceived the virtual robot in general as more credible than German native speakers. There was no significant main effect of the virtual robot’s accent (*F*(1, 209) = 1.49, *p* = .223, $\eta_{p}^{2}=.01$ ). The interaction was significant (*F*(1, 209) = 11.13, *p* = .001, $\eta_{p}^{2}=.05$ ).

For sympathy we found a significant main effect of participants’ mother tongue (*F*(1, 209) = 21.41, *p* < .001, $\eta_{p}^{2}=.07$ ). English native speakers perceived the virtual robot in general as more sympathetic than German native speakers. The virtual robot’s accent showed no significant main effect (*F*(1, 209) = 0.50, *p* = .479, $\eta_{p}^{2}=.00$ ) and there was no significant interaction (*F*(1, 209) = 1.99, *p* = .160, $\eta_{p}^{2}=.01$ ).

## 5 Discussion

This study was conducted to explore the effects of a virtual robot with German-accented English, regarding the perception of the robot’s personality as well as the dimensions perceived warmth and competence. This was done in an effort to further investigate the perception of non-native accents to contribute to the research surrounding enculturated SIAs.

Contrary to our expectations, the German native speakers did not rate the virtual robot with the German accent as warmer, more competent and higher regarding sympathy, even though the virtual robot’s accent was recognised as German. Therefore, H1 has to be rejected.

The results even showed significantly higher perceived competence for the standard English condition compared to the condition where the virtual robot was speaking German-accented English. Participants might have attributed the less fluent speech of the virtual robot, speaking with a German accent to a technical shortcoming rather than a cultural cue. Furthermore, the lower perceived competence of the virtual robot speaking with a German accent could be due to lower perceived language proficiency of the virtual robot regarding the English language.

This does not resemble the effects of the similarity attraction principle, however it falls in line with previous research with SIAs, that suggests that users prefer an agent speaking with a prestigious accent (see Aylett et al., 2021 for an overview).

The results also revealed that the virtual robot speaking with German-accented English was not generally rated significantly higher on the scales credibility and conscientiousness. However, within the English native speakers the virtual robot speaking with German-accented English was rated as being significantly more credible. Hence, H2 can be partly accepted. This means some German stereotypes could be triggered within English native speakers by watching a virtual robot that speaks with a synthetically generated German accent. The virtual robot speaking with German-accented English did not trigger German stereotypes within German native speakers. However, stereotypes are usually identified by evaluating how outgroups perceive each other rather than how a certain group perceives its own members. Therefore, Germans might not generally perceive themselves as *most hard working* or *most trustworthy*. In addition to this, previous research has shown that the cultural background of a social robot’s recipient can have an impact on the perception of this robot (see Lim et al., 2021 for an overview). The results of a study conducted by Eimler et al. (2011) for example revealed differences in how German and United States American participants perceived emotion in social robots. This could also explain, why German native speakers perceived the virtual robot differently than English native speakers. Regarding our research question, whether the virtual robot’s accent affects its perceived personality regarding *extroversion, agreeableness, neuroticism, conscientiousness* and *openness*, we found that the virtual robot speaking with German-accented English was generally rated as being more neurotic. This could be caused by the fact that the synthetic German accent was not as fluent as the standard English which could have resulted in the virtual robot appearing as less self confident or less emotionally stable. These results are also in line with previous research by Zárate-Sández (2017) which shows that neuroticism acts as a reliable predictor for a person retaining a strong non-native accent when learning a new language. Our results suggest that recipients of a non-native accent also attribute the respective personality traits to the speaker that have been shown to act as a predictor for the accent itself.

The results revealed that English native speakers rated the virtual robot higher on the dimensions *warmth, competence, credibility,* and *sympathy* compared to the German native speakers. This is a clear cross cultural effect on the perception of SIAs in general. However, because the virtual robot spoke English in both conditions, this might have positively impacted its rating by English native speakers, possibly triggering an ingroup effect compared to the German native speakers (Turner et al., 1987; Fiske, 2018).

When asking the participants how well they could understand the virtual robot, we found that regardless of their mother tongue, participants reported higher intelligibility when they watched the video with the virtual robot speaking in standard English. This is in line with previous research that reports lower intelligibility when listening to non-native accents (Derwing and Munro, 2009; Munro, 2003). Furthermore, the synthetically generated German accent has proven to work as both, a general marker as a non-native speaker, as well as a specific marker as a German native speaker, since the majority of the participants recognised the virtual robot speaking with German accented English as a German native speaker.

### 5.1 Recommendations for enculturated SIAs

Generally, the virtual robot was perceived more positively by the English speaking participants, which suggests a strong connection of the participants’ mother tongue and the SIAs implemented mother tongue. This could mean that the effect of the virtual robot’s language was stronger than the effect of its accent. Therefore, it could be beneficial to adopt a SIA’s language to the mother tongue of its user, even if the user is multilingual.

For triggering the impression of a mixed-cultural English speaking virtual robot with a German background, the cultural cue of a synthetically generated German accent has proven to be sufficient, since most of the participants were able to correctly identify the accent.

When not only the impression of a mixed-cultural SIA but also the activation of the respective stereotype is desired, other culture-specific cues in addition to the non-native accent should be considered, since the synthetic accent alone only partially triggered German stereotypes in English native speakers and none in German speakers. This also suggests that it is easier to trigger stereotypes in recipients of a different culture than the one implemented. Therefore, the design of enculturated SIAs should be thoroughly attuned to the cultural background of the recipient, if the goal is to trigger certain stereotypes. When the aim is to trigger an ingroup effect using a mixed-cultural SIA by solely adapting its speech, designers should bear in mind that the language the SIA speaks might have a bigger effect than the non-native accent. Again, adding visual cultural cues or attuning the SIA more specifically to the user’s cultural background might also help in triggering the feeling of a social ingroup, despite the language that is being used.

Designers of enculturated SIAs should be aware that while they might be able to trigger the impression of a mixed-cultural SIA by implementing a non-native accent, this accent can also lead to lower intelligibility, as well as the impression of the SIA to be more neurotic and less competent. Therefore, the implementation of culture-specific cues in form of non-native accents should be considered carefully, since it might lead to unwanted side effects.

## 6 Conclusion and future work

This paper provided an overview of the research conducted on mixed-cultural SIAs and presented a preliminary study on the impact of a non-native German accent on the perception of an English speaking virtual robot, as reported by German and English native speakers. The participants could reliably identify the virtual robot with the German accent as a German native speaker. The virtual robot with the German accent, however, was only partially able to trigger German stereotypes and only within English native speakers. Furthermore, the effect of the virtual robot’s native language has proven to be of high relevance, as the virtual robot was generally perceived more positively by English native speakers. The virtual robot’s German accent did not trigger the expected ingroup associations within the German native speakers. The conducted study also revealed possible negative effects of using synthetic non-native accents such as lower intelligibility, and the perception of the virtual robot to be more neurotic and less competent. These findings offer valuable insights in the perception of mixed-cultural SIAs and can be used to improve the design of enculturated agents.

In our future work we will further investigate the effect of non-native accents with SIAs. After conducting the first study using a virtual robot, we will conduct future studies using physical robots to see, how social robots with non-native accents are perceived. Furthermore, we will investigate how the perception of mixed-cultural SIAs changes if other modalities like culture-specific appearance and non-verbal behaviour are implemented in addition to the non-native accent. Further research in this area will help to design convincing mixed-cultural SIAs and set the base for self-reflective culturally aware SIAs, that either automatically adapt to the user’s cultural background, or purposefully don’t adapt depending on the intended outcome.

## Data Availability

The datasets presented in this article are not readily available because the authors are withholding the data for further calculations. Requests to access the datasets should be directed to the corresponding author.

## Footnotes

1
https://www.infosim.net/

2
https://www.blender.org/

3
https://unity.com/

4
https://aws.amazon.com/de/polly/

5
https://www.prolific.co/
